# Visualization of hot flows of tall space fires in model experiments with Schlieren Photography technique

**DOI:** 10.1186/s40064-016-3422-8

**Published:** 2016-10-07

**Authors:** ChungHwei Su, ShiuanCheng Wang, ChiaYuan Shih, YungChang Yang

**Affiliations:** Department of Safety, Health and Environmental Engineering, National Kaohsiung First University of Science and Technology, No. 1, Daxue Rd., Yanchao Dist., Kaohsiung City, 82445 Taiwan

**Keywords:** Tall space, Natural smoke exhaust system, Schlieren Photography technique, Fire dynamics simulator (FDS)

## Abstract

The natural smoke exhaust system for tall spaces is more advantageous than the mechanical type of exhaust. In Taiwan, the effectiveness of natural smoke exhaust systems is inspected only by checking the vent area size. However, the air flow field in a tall space is very complicated, both at ordinary times or during fires. This study used Schlieren Photography technique, on the principle that light rays are refracted when penetrating materials of different densities, to test and simulate the dynamic measurement of hot air in tall space model. A single-mirror Schlieren system, including an 838 mm (H) × 736 mm (W) square concave mirror, as well as the focal length of 3100 mm was adopted. The experimental process of six smokeless candles were used for 1/12.5 model experiment to record the dynamic distribution and accumulation of air flow in the abovementioned space. FDS software was used to simulate various fire scenarios. The different locations of openings in some cases were studied with the maximum temperature scales of 40 and 45 °C, separately. The simulation results and experimental images showed highly similar hot air flow patterns. Schlieren Photography was proved capable of recording and visualizing the dynamic flow of hot air immediately, directly and accurately.

## Background

### Flow characteristics of tall spaces

**Fig. 1:**
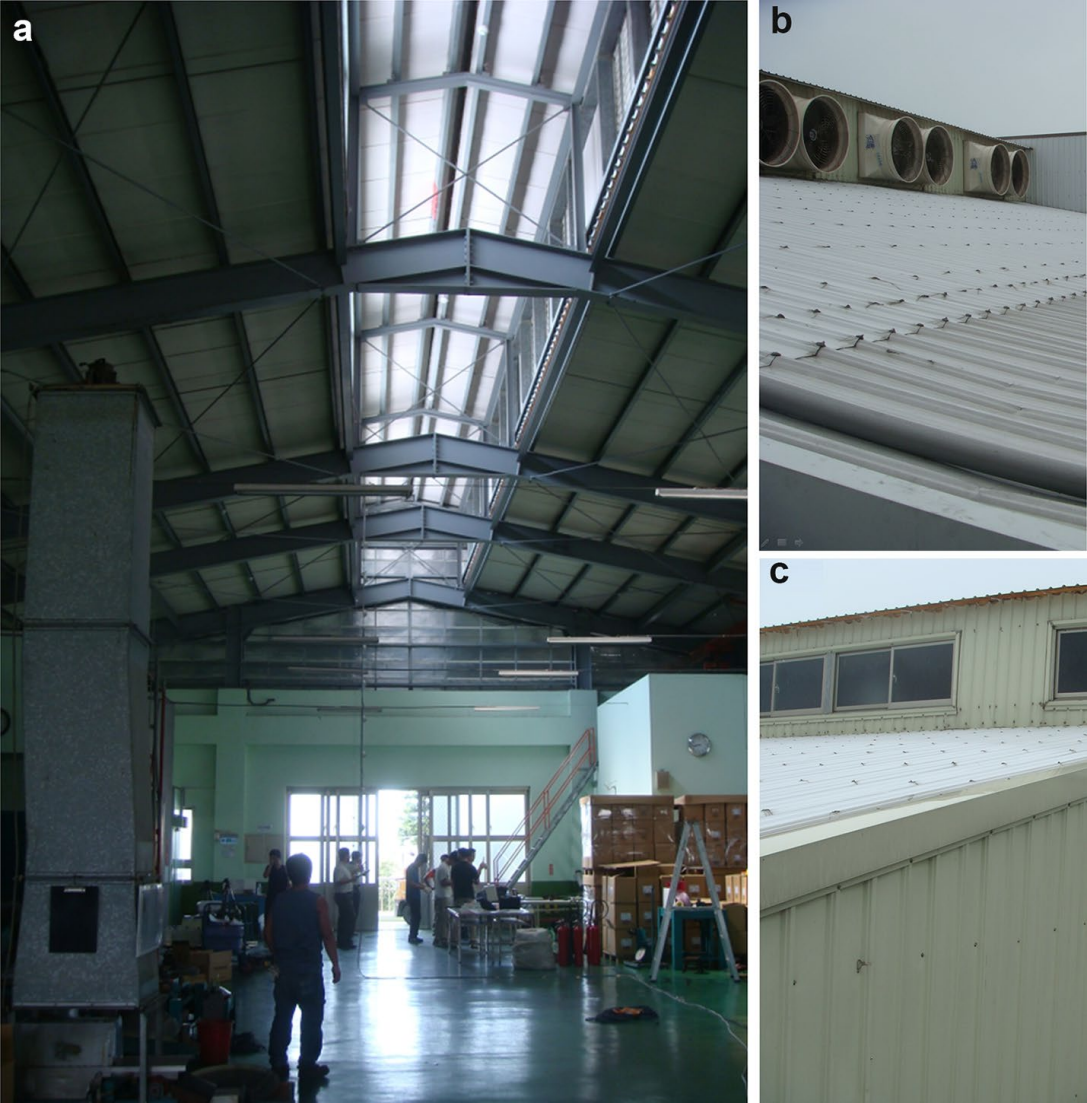
**a** The interior space in a tall space. **b** Mechanical ventilation equipment in this tall space. **c** Natural ventilation windows in this tall space

Some factories need a tall space for product features or process, such as mechanical equipment assembly industry, glass finishing industry or metal processing industry. A tall space has a high rooftop and a large span, as shown in Fig. 1a, and the internal flow pattern is relatively complicated. With multiple heat sources, such as equipment on the ground or sunshine, the air flow stratification phenomenon sometimes occurs in these spaces (Lau and Niu 2003; Cheng et al. 2015).

Since Taiwan is hot in summer, many factories are equipped with air conditioning or ventilation equipment. Figure 1b, c illustrate the mechanical and natural ventilation equipment for factories. Some studies conducted numerical analysis to discuss the distribution of air flow and temperature field (Ramponi and Blocken 2012; Niamsuwan et al. 2015). When a fire happens, the stack effect would be generated in tall factories. The make-up effect also influences the flow of hot air and the distribution of smoke (Gutiérrez-Montes et al. 2010; Su et al. 2011; Chen et al. 2015). The analysis of air flow in the tall spaces, whether at ordinary times or during fires, is an important topic (Chow 1998; Li et al. 2004; Ayala et al. 2016).

### The natural smoke exhaust system

The smoke at the fire scene is one of the major causes of death. Effective smoke extraction must be planned in constructing industrial factory buildings. In recent years, the manufacturing industry has gradually been paying greater attention to energy saving; the concept of using the thermal buoyancy effect of air flow for natural ventilation has been integrated in many tall buildings (Evola and Popov 2006; van Hooff and Blocken 2012). When a fire happens, the natural ventilation can be converted into natural smoke extraction.

The natural smoke exhaust system has many advantages, including low cost, no power and low maintenance cost (Huo et al. 2004; Sun et al. 2013). According to *The Standard for Installation of Fire Safety Equipment Based on Use and Occupancy*, the area of natural smoke ventilation needs to be over 2 % of the smoke compartment area, and the smoke needs to be discharged to the outside naturally. According to fire codes, buildings have to be equipped with smoke exhaust fans if the smoke cannot be discharged naturally (National Fire Agency 2012).

### Purpose of the study

In Taiwan, the proper functioning of natural smoke exhaust systems is inspected only by checking whether the opening area meets the specification. Since the smoke is exhausted by air flow, the natural smoke exhaust system can effectively control the smoke if the flow pattern of hot air at the fire scene is analyzed clearly. The numerical simulation is the common international method for research on airflow pattern (Deckers et al. 2013; Weng et al. 2014; Su et al. 2015). Besides numerical simulation, Schlieren Photography was adopted to record the flow pattern. The simulation result of the famous fire simulation software, fire dynamics simulator (FDS), was compared with the Schlieren image; in addition, the hot air movement was effectively visualized and quantified. Different maximum temperature scales were compared one by one, and the display of the flow pattern of hot air after burning was optimized in this paper.

## Methods

### Scaling model in fires

The correct solution cannot be obtained from theories since the combustion phenomenon is quite complicated; like many phenomena, it still need to be validated by experiments. If the flow field can be confirmed by the deduced empirical formula and the mathematically derived equations at a low cost and within a reasonably short time, the fluid flow problem in engineering can be solved (Kuwana et al. 2013; Kim and Rie 2016).

In consideration of the space and cost factors, the full scale is hardly tested. The laboratory uses Similarity Law and models to test fluid flow. Table 1 shows the equation of the scale law (Quintiere et al. 1978). The result will be distorted if the model is extremely small; in consideration of accuracy, this study uses a 1:12.5 model to test the experimental subject.

**Table 1:** The relationship of the parameters between the model and prototype

Physical properties	Proportion^a^
Geometry	*X*_*m*_ = *X*_*f*_(*l*_*m*_/*l*_*f*_ *)*
Velocity	*V*_*m*_ = *V*_*f*_(*l*_*m*_/*l*_*f*_ *)* ^1/2^
Time	*t*_*m*_ = *t*_*f*_(*l*_*m*_/*l*_*f*_ *)* ^1/2^
Temperature	*T*_*m*=_ *T*_*f*_
Heatrelease rate	*Q*_*m*_ = *Q*_*f*_(*l*_*m*_/*l*_*f*_ *)* ^5/2^

^a^The proportion between the model and the full prototype is *l*_*m*_:*l*_*f*_

### Numerical simulation method

A fire dynamics simulator (FDS) was used in this study. It was developed by Building and Fire Research Laboratory, National Institute of Standards and Technology (NIST), using governing equations of low Mach numbers to describe the airflow phenomenon. The FDS simulates the three-dimensional buoyancy-driven airflow at the fire scene based on Large Eddy Simulation (LES). The framework of FDS is divided into three phases: pre-process, numerical computation solver and post-process (Kerber and Milke 2007; Goldsworthy 2012).

The fluid velocity, temperature, density and pressure were calculated by the energy equation, the momentum equation, other equations and the spatial average of total pressure equations of temperature, density and pressure. The time derivative term needs to be discretized from the second-step of the Runge–Kutta method. The conservation equations for mass, momentum and energy for a Newtonian fluid are as shown in Eqs. (1–4) (Wang et al. 2011; McGrattan et al. 2015):

- Conservation of mass:
  1 $$\frac{\partial \rho }{{\partial {\text{t}}}} + \nabla \cdot \rho \,{\text{u}} = {\dot{\text{m}}}_{\text{b}}^{'''}$$
- Conservation of momentum (Newton’s Second Law)
  2 $$\frac{\partial }{{\partial {\text{t}}}}\left( {\rho \,{\text{u}}} \right) + \nabla \cdot \rho \,{\text{u}}\,{\text{u}} + \nabla {\text{p}} = \rho {\text{g}} + {\text{f}}_{\text{b}} + \nabla \cdot \tau_{ij}$$
- Conservation of energy (First Law of Thermodynamics)
  3 $$\frac{\partial }{{\partial {\text{t}}}}\left( {\rho \,{\text{h}}} \right) + \nabla \cdot \rho \,h\,{\text{u}} = \frac{Dp}{Dt} + {\dot{\text{q}}}^{'''} - {\dot{\text{q}}}_{\text{b}}^{'''} - \nabla \cdot {\dot{\text{q}}}^{\prime \prime } + \varepsilon$$
- Equation of state for a perfect gas
  4 $$p = \frac{\rho RT}{{{\bar{\text{W}}}}}$$

Herein, ρ: density (kg/m^3^); fb: external force vector (N/m^3^); g: gravity vector (m/s^2^); h: sensible enthalpy (kJ); p: pressure (pa); R: universal gas constant (J/K mol); T: temperature (°C); u: velocity vector u(u, v, w) (m/s); ${\dot{\text{m}}}_{\text{b}}^{'''}$: mass production rate per unit volume (kg/s m^3^); ${\dot{\text{q}}}^{'''}$: heat release rate per unit volume (kg/s m^3^); ${\dot{\text{q}}}_{\text{b}}^{'''}$: energy transferred (kg/s m^3^); ${\dot{\text{q}}}^{{\prime \prime }}$: heat flux vector (J/s m^2^); $\varepsilon$: dissipation rate (kg/s m^3^); $\tau_{{ij}}$: viscous stress tensor (N/m^2^); ${\bar{\text{W}}}$: molecular weight of the gas mixture (g/mol).

The grid setting must take into consideration both the calculation speed and accuracy. The two items are the main considerations in the simulation process (Tu et al. 2012; Zhu et al. 2015; Su et al. 2011; Sun et al. 2013). This study uses the Characteristic Fire Diameter to evaluate the optimum grid size, and analyzes the optimum grid size at a specific heat release rate. McCaffery proposed using the minimum length scale of fire plume as the characteristic fire diameter D* to determine the grid size (Baum and McCaffrey 1989), expressed as Eq. (5):

5 $$D^{*} = \left[\frac{{\dot{Q}}}{{\rho_{\infty } \,C_{\infty } \,T_{\infty } \,\sqrt g }}\right]^{2/5}$$

where $\dot{Q}$: total heat release rate (kW); $\rho_{\infty }$: Air density (kg/m^3^); $C_{\infty }$: Air specific heat (kJ/kg K); $T_{\infty }$: Space temperature (K); g: Gravitational acceleration (m/s^2^).

Only if the grid size is about 1/10 the characteristic diameter (D*), will the time average axis velocity and temperature calculated by the LES method match the experimental regression equation of McCaffery.

### Schlieren Photography

Optical visualizing technology is a common non-invasive technology for fluid visualization experiments. The light rays are refracted when penetrating media of different densities as the refractive index is changed. At present, common studies use computer simulation for analysis; some experiments use smoke as trace particles to observe the air flow indirectly (Xiao 2012; Li et al. 2014).

The optical imaging techniques include the shadowgraph method, Schlieren Photography and optic holography (Panigrahi and Muralidhar 2012). In 1864, August Toepler et al. used instruments to obtain black and white picture of one-dimensional flow field. In 1981, G.S. Settles replaced the cutter blade by the color filter of RGB color wheel to generate color images (Neumann and Ermert 2006; Settles 1981). In 1999, Mercier and Jaluria used a model and Schlieren Photography to study the heat convection and heat transfer at a staircase warehouse and house gate on fire (Mercier and Jaluria 1999).

The relationship between the gas density, ρ, and the gas refractive index, n, can be represented by the Gladstone–Dale equation, as in Eq. (1) (Schwarz 1996; Martínez-González et al. 2012; Su and Bai 2016):

6 $$\frac{n - 1}{\rho } = G\left( \lambda \right)$$

where G(λ) is defined for air by:

7 $$G\left( \lambda \right) = 2.2244 \times 10^{ - 4} \left[ {1 + \left( {\frac{{6.7132 \times 10^{ - 8} }}{\lambda }} \right)^{2} } \right]$$

8 $$\Delta h = \frac{{l_{b} f}}{{l_{b} + l_{c} - f}} \times \frac{1}{n}\,\int\limits_{{l_{b} - \Delta l_{b} }}^{{l_{b} + \Delta l_{b} }} {\frac{{\partial n}}{{\partial r}}} dl$$

Here in n: gas refractive index; ρ: the gas density (kg/m^3^); G(λ): Gladstone-Dale number(m^3^/kg); Δh: the offset distance (m); f: lens focal length (m); λ: wave length (m).

**Fig. 2:**
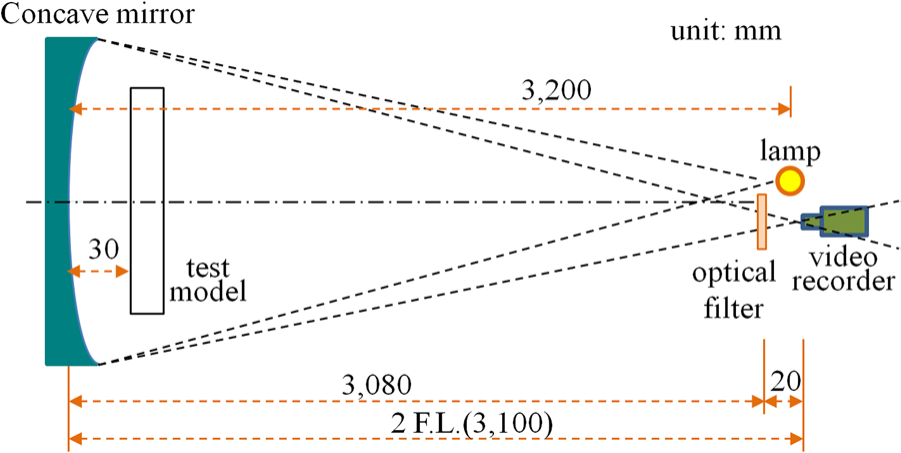
The single-mirror coincident Schlieren Photography system

Schlieren Photography is divided into single-mirror type and multi-mirror type. This study uses single-mirror Schlieren Photography; the principle is shown in Fig. 2.

## Experimental equipment and numerical simulation conditions

### Description of the apparatus

**Fig. 3:**
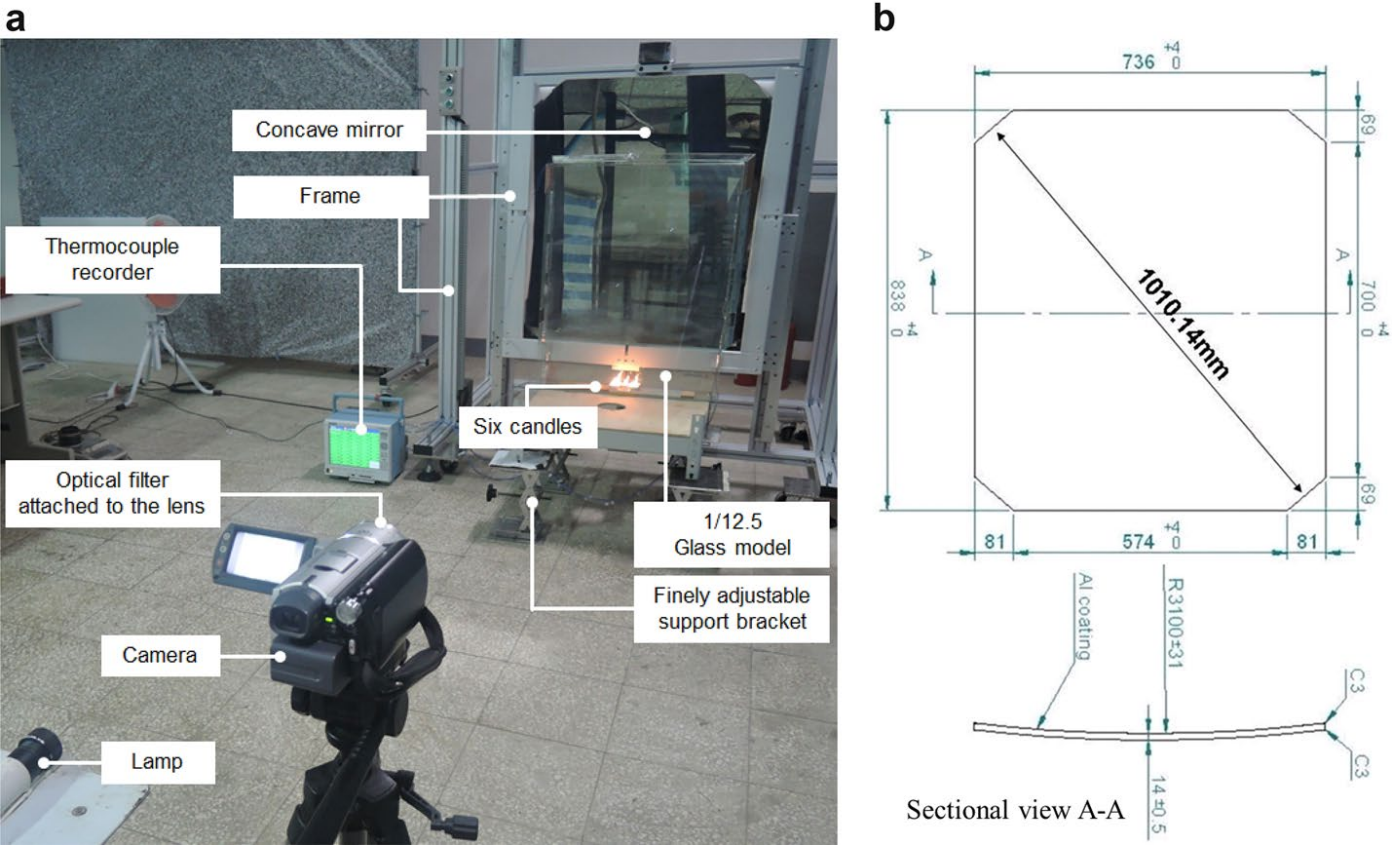
The description of experimental devices. **a** Device configuration. **b** The dimensions of the concave mirror

The lamp was set at the same height with the concave mirror center, and the light source emitted towards the spherical mirror. The numbers of smokeless candles were used to control the size of fire source and the Color Schlieren for observing the variations of hot air flow in the scaled-down model. The equipment configuration used in this study is shown in Fig. 3a, b, described below:

1. Led light source: the LED light source is covered, leaving a small hole with a diameter of 1 mm as the fixed light source.
2. Smokeless candle: a smokeless candle is used as the fire source. The Cone Calorimeter is used for measurement in the burning process, as shown in Fig. 4. After a 1200-s test, the heat release rate of each candle is 0.027 kW.

**Fig. 4:**
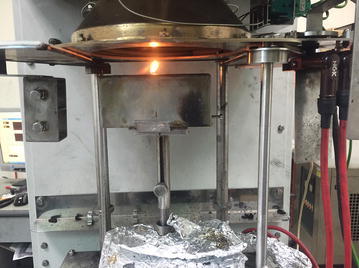
The measurement condition of the smokeless candle in the cone calorimeter
3. Square concave mirror: the size is 736 mm (W) × 838 mm (H). The focal length is 3100 mm, as shown in Fig. 3b. It is made by optical instrument manufacturer in Taiwan, for focusing the reflected parallel lights from light source to a point. The plated film is aluminum and SiO protective film.
4. Color filter: the color filter displays the changes in the image colors. By comparison imaging results, they displayed that brown filter was better than the other colors.
5. Digital camera: SONY DCR-SR100 HDD type digital camera with a 1/3″, 3.3 megapixel advanced HAD CCD imager in the experiment, the image equipment for shooting is the Schlieren.
6. Temperature measuring equipment: the thermocouple measures the temperature changes at three points: T1, T2 and T3 points.
7. Wood board: for closing the opening, so that the glass model has changing combinations.

### Experimental model geometry

**Fig. 5:**
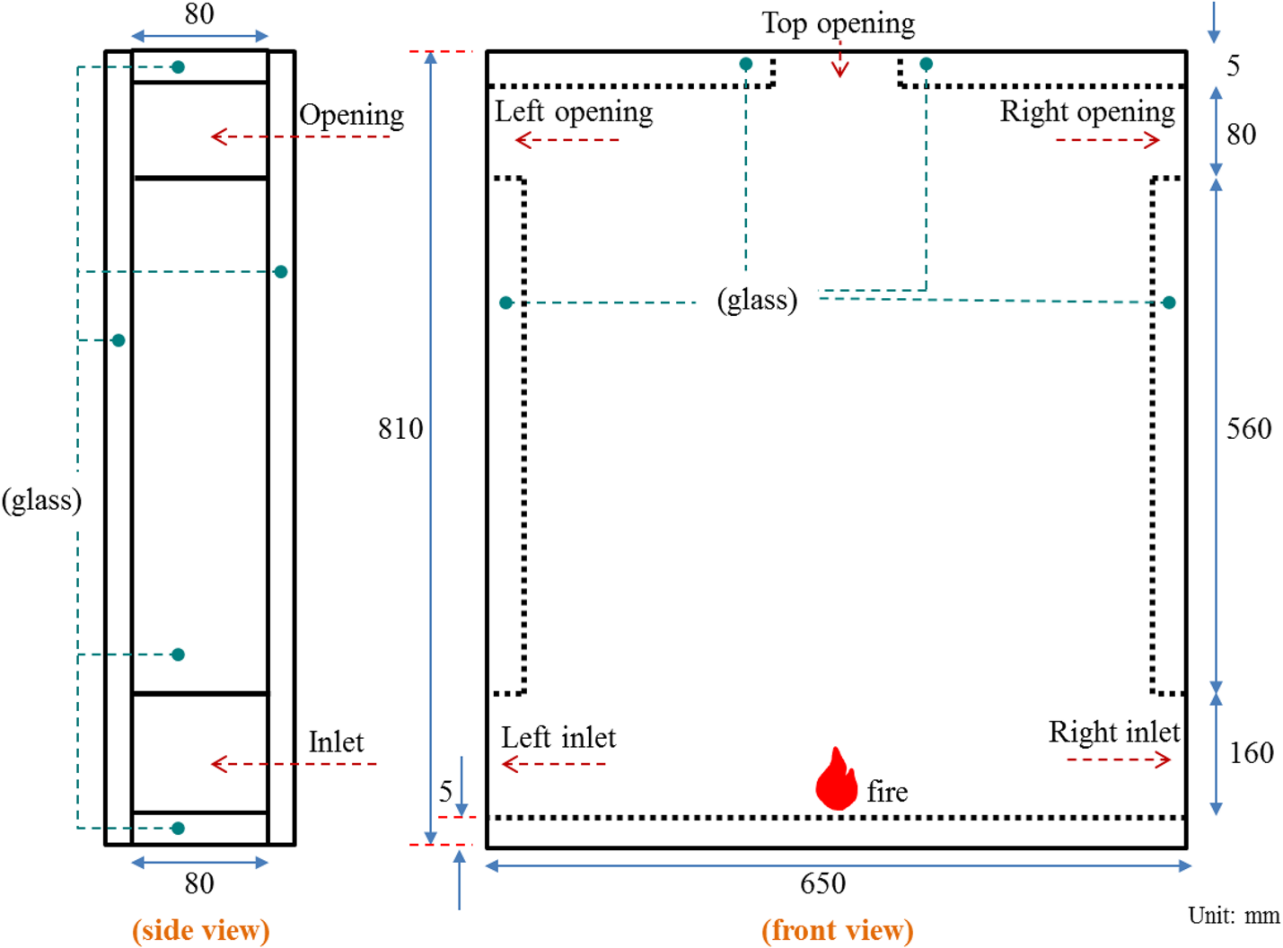
The dimensions of experimental model

This model is composed of 5 mm thick glass. The inner space is 0.64 m (width) × 0.8 m (height) × 0.08 m (depth). Considering the airtightness, all the glass is cemented by silica gel to avoid hot air leakage. The upper and lower openings are opened according to the experimental requirements; the opening is sealed with wood board, as shown in Fig. 5. The dimensions of simulation and experimental models are shown in Table 2. The symbols of various cases are described in Table 3. The thermocouple line is mounted for temperature measurement. The thermocouple line is kept away from the glass wall in the experimental process to avoid measurement errors.

**Table 2:** The dimensions of the prototype and experimental model

	Prototype (m)	Model (m)
Height	10	0.80
Width	8	0.64
Height of the inlet	2	0.16
Height of the opening	1	0.08

Scale: 1/12.5

**Table 3:** The symbol description of cases

Symbol	Description
O	Opening for smoke extraction
I	Inlet for make-up air
L	Left side
R	Right side

### Simulation conditions

The available concave mirror size for this study is 736 mm (W) × 838 mm (H). Since the visualization region must be reserved to shoot the flow behavior of hot air from the model, the model width is 640 mm, and the height is set as 800 mm. Referring to the scale value, the model for executing FDS is set as 8 m (width) × 10 m (height) × 1 m (depth). The space is 8 m wide and 10 m high to meet the definition of tall space.

There are about 2.3 million cells. The fire point is in the model center; the combustible material is assumed to be polyurethane. The heat release rate of combustion is set as 89.5 kW (= Six candles × 0.027 kW × $12.5^{5/2}$). The mass burning rate is to reach the maximum heat release value immediately after ignition, so as to meet the characteristic of candle burning.

## Results and discussion

### Comparison of experimental measurements and simulation results

**Fig. 6:**
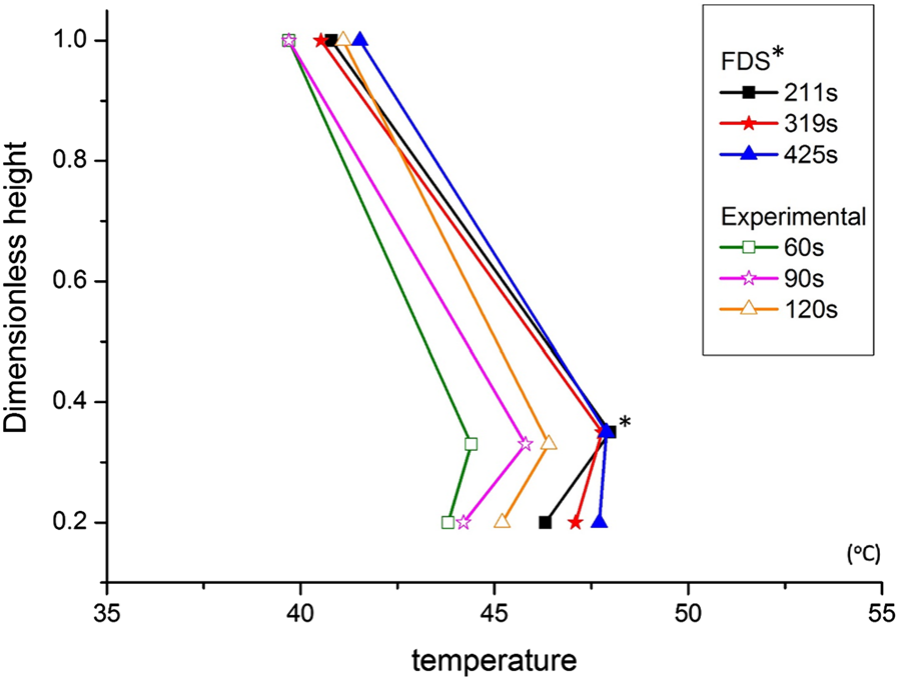
Experimental and simulated temperature profiles for Case OR_IL. *Higher central point in the FDS simulation: the location of the measuring point need exactly fit to the multiple of the grid dimension

Since the combustion of candles in a model will produce hot air, as well as high measured temperatures at holes indicated the hot air flowed out the model, the direction of the hot airflow in model was able to be observed by measured values of the temperatures. The burning process of Case OR_IL was recorded first. The changes in the temperature field in the simulated space and experimental model were analyzed. Figure 6 shows the temperature variation of the experimental model at different times. After the assessment of the thermocouple characteristic (transmission rate), measured temperature were calculated using the average values within 3 s. The times recorded were 60, 90 and 120 s, respectively. The measurement positions were in front of the right wall with the ratio of relative height 0.2, 0.33 and 1, respectively. Figure 6 also shows the simulation temperature in the FDS software. Since the scale was 1:12.5, the recording times were the ratio of $\sqrt {12.5}$ times, which were 212, 319 and 425 s, respectively. The external temperatures for the experiment and simulation were 20.5 and 20 °C, as shown in Table 4.

**Table 4:** The comparsion of experimental results with the simulation temperatures

Height	Time (s)		
	Simulation		
	212^a^	319	425
T3	40.8 (20.3)^b^	40.5 (20.0)	41.5 (21.0)
T2	48.0 (27.5)	47.8 (27.3)	47.9 (27.4)
T1	46.3 (25.8)	47.1 (26.6)	47.7 (27.2)

Height	Time (s)		
	Experiment		
	60	90	120
T3	39.7 (19.7)	39.7 (19.7)	41.1 (21.1)
T2	44.4 (24.4)	45.8 (25.8)	46.4 (26.4)
T1	43.8 (23.8)	44.2 (24.2)	45.2 (25.2)

^a^Time scale: $$1/\sqrt {12.5}$$

^b^The temperature difference from initial temperature: experiment: 20.5 °C/simulation: 20 °C)

**Fig. 7:**
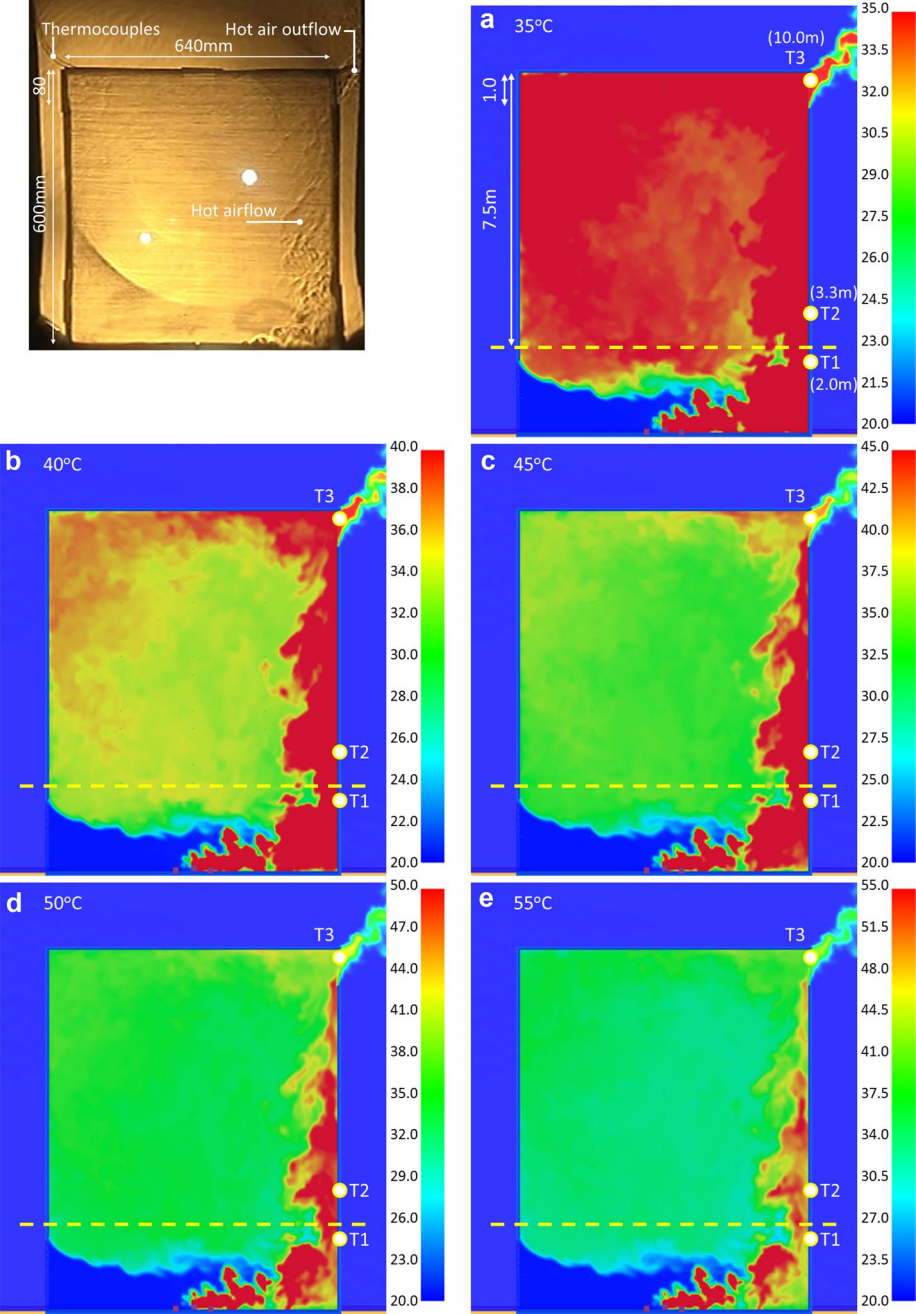
The comparison of the Schlieren image and simulation results with various temperature scales for CASE OR_IL (@60 s)

According to Fig. 6, the experimental results and simulated values exhibited very close trends. The locations of measuring points T1, T2 and T3 are shown in Fig. 7. According to Table 4, in the model experiment, the temperatures at T1, T2 and T3 at 60 s were 43.8, 44.4 and 39.7 °C, respectively. The temperatures rose to 44.2, 45.8 and 39.7 °C at 90 s. The temperatures were 45.2, 46.4 and 41.1 °C at 120 s. Compared with the numerical simulation results, the temperatures at T1, T2 and T3 were 46.3, 48.0 and 40.8 °C at 212 s, respectively. The temperatures rose to 47.1, 47.8 and 40.5 °C at 319 s. Furthermore, the temperatures were 47.7, 47.9 and 41.5 °C at 425 s.

The results show that the two temperatures were similar at the upper outlet T3. The difference between the temperature increments was within 1 °C. However, at the lower T1 and T2, the temperature values of the simulation were higher; perhaps due to the glass in the positions heat sinking, the experimental temperatures were lower. The assumption in simulation processes of FDS did not consider the heat dissipation of hot air through the wall, so the temperatures were relatively high.

### Optimal display in simulation images

The concave mirror for the experiment is 736 mm (W) × 838 mm (H). The model size was constructed in 640 mm (W) × 800 mm (H) to get better Schlieren images. Therefore, the model height was set as focusing on the region at upper 60 cm of the model, as the region above the yellow dotted line marked in Fig. 7a–e. The simulation results of Case OR_IL are displayed by 5 maximum temperatures: 35, 40, 45, 50 and 55 °C; the optimum display effect was analyzed. The criterion of judgment was the complete presentation of flow condition of hot air. Three time points were compared, as described below:

#### Results comparison of Case OR_IL at 60 s

Figure 7 shows the image of hot air distribution at 60 s during the experiment. The simulation states of the hot air flow at five different maximum temperature scales were compared at 212 s. Figure 7a shows the air flow distribution when the maximum temperature was 35 °C. The simulation result displayed in the region above the yellow dotted line was a sheet of red, meaning the temperatures were higher than 35 °C. It was observed that the outside airflow was obviously imported from the lower left of the model. The hot air outflow in the upper right region was also obviously evident. However, the overall evaluation shows that the flow pattern of the simulation result was dissimilar from the Schlieren image of hot air in the experiment.

Figure 7b, c show the simulated air flow distribution when the maximum temperatures were 40 and 45 °C, respectively. The fluid flows in from the lower left, and flows out from the upper right. The upper part above the yellow dotted line shows the right side of space as red, meaning the hot air of combustion accumulates near the right wall. There is clear hot air in the upper left region of Fig. 7b. Compared with the air flow in the Schlieren image, the simulation result was closest to the experimental record when the maximum temperature scale was 40 to 45 °C. If the maximum temperature scale was 40 °C, the high-temperature region of dynamic image was displayed clearly. When the scale was 45 °C, the high-temperature region of static picture was displayed well.

Figure 7d, e show the hot air distribution when the maximum temperature scales were 50 and 55 °C, respectively. Differing from previous results, there was less red region on the right side. The regimes of the hot air flow were compared with the Schlieren results: the temperature states were dissimilar.

#### Results comparison of Case OR_IL at 90 s

**Fig. 8:**
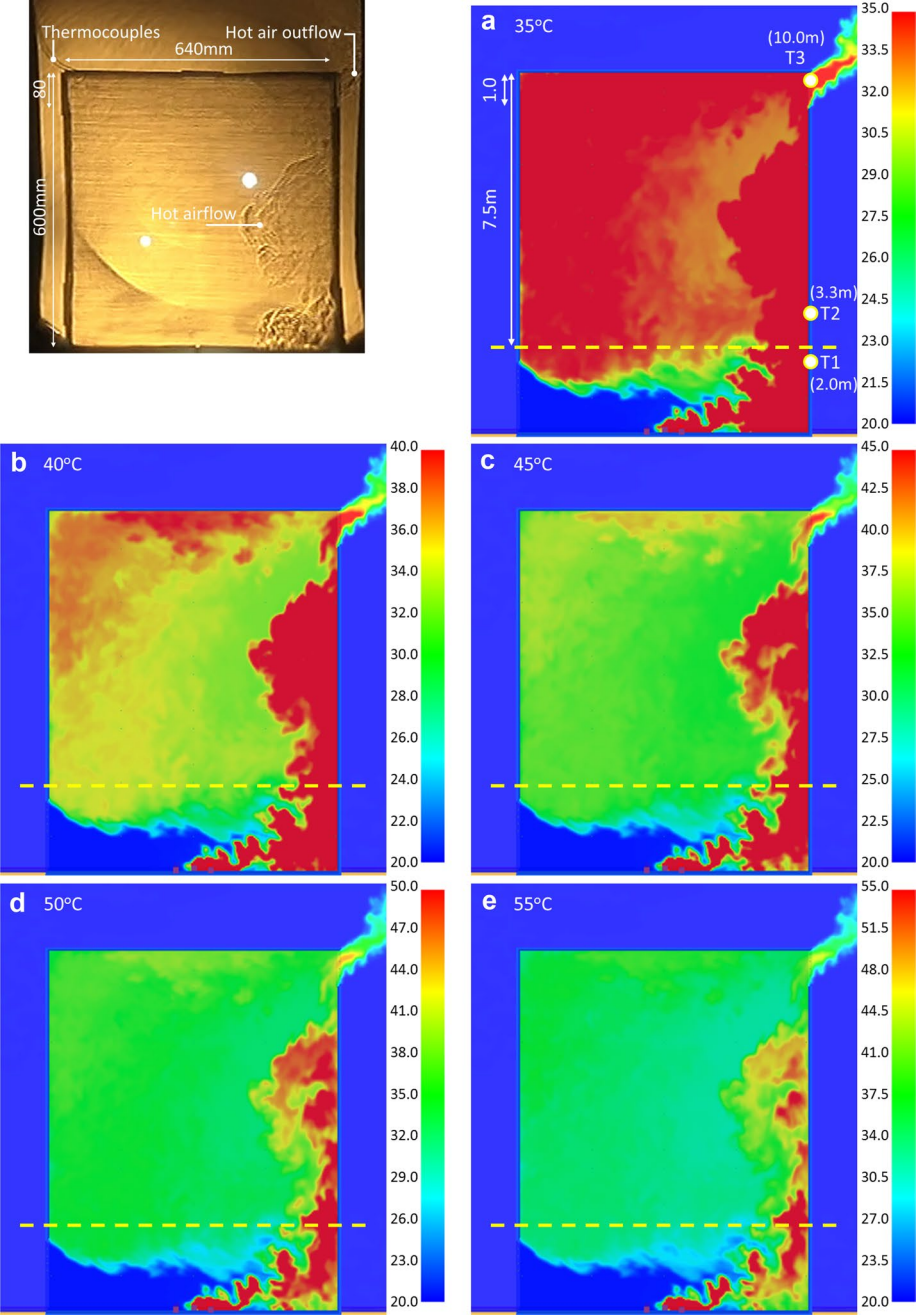
The comparison of the Schlieren image and simulation results with various temperature scales for CASE OR_IL (@90 s)

Figure 8 shows the images of hot air at 90 s of the experiment and at 319 s of the simulation. Figure 8a shows the flow regime when the maximum temperature was 35 °C. The same as Fig. 7, the space was all red. But the experimental and simulation results differ. Figure 8b, c show the flow patterns at 40 and 45 °C. The inflow and outflow of hot air were similar to the experimental results. There were more red regions than in Fig. 7, meaning the heat accumulated gradually and the temperatures rose gradually. This trends were similar to Fig. 6. The results show that the simulation and experimental results were closest to each other when the maximum temperature was 40 to 45 °C. According to the matching results, the dynamic demonstration are displayed clearly when the maximum temperature scale was 40 °C. The temperature of 45 °C was suitable for static pictures.

Figure 8d, e show the hot air distribution when the temperature scales were 50 and 55 °C, respectively. There were fewer red regions. Since the maximum temperature was relatively high, the air flow at the upper right outlet cannot be displayed completely.

#### Results comparison of Case OR_IL at 120 s

**Fig. 9:**
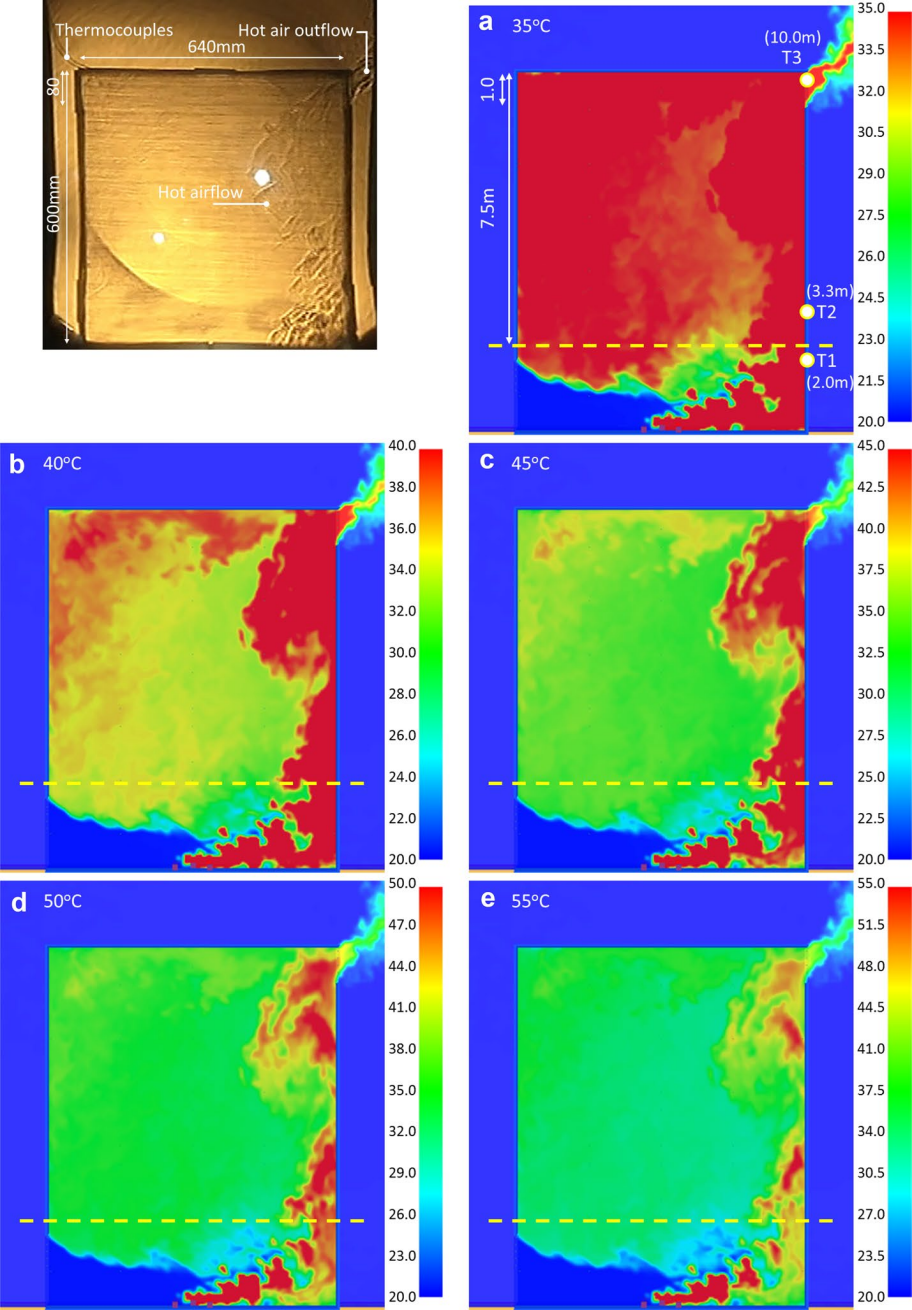
The comparison of the Schlieren image and simulation results with various temperature scales for CASE OR_IL (@120 s)

The hot air images at 120 s of the experiment and at 425 s of the simulation are compared in Fig. 9. The results show that when the maximum temperature scales were 40 and 45 °C in Fig. 9b, c, the simulation results matched the images of the Schlieren experiments. The former was applicable to dynamic images; the latter one indicated a static picture. The raised temperature was similar to Fig. 6. The simulation results of Fig. 9a, d, e cannot match the images of the Schlieren experiments.

### Verification of other cases

In order to prove that the more appropriate range of maximum temperature indicators is from 40 to 45 °C, which is suitable for a static picture and a dynamic demonstration, the simulation results are verified by Case OL_IL and Case OT_IL:

#### Schlieren images and simulation results of Case OL_IL

**Fig. 10:**
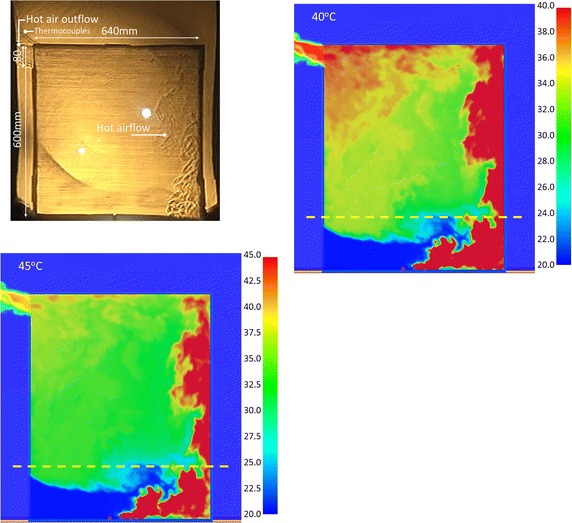
The verification of the Schlieren image and simulation results for CASE OL_IL at 90 s

**Fig. 11:**
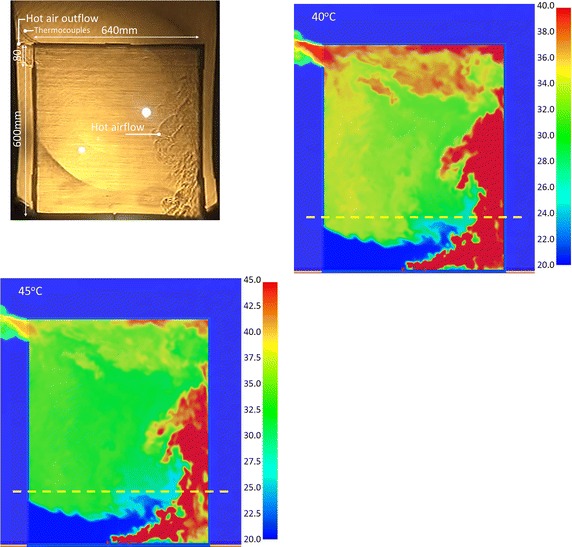
The verification of the Schlieren image and simulation results for CASE OL_IL at 120 s

Figures 10 and 11 show the Schlieren images at 90 and 120 s in Case OL_IL for comparison with the simulated hot air flow pattern at 319 and 425 s. The maximum temperature scales were set as 40 and 45 °C. The two patterns illustrate that the hot air distributions were very similar. At 90 s, Fig. 10 shows the simulation result: the external air entered from the lower left, so the hot air of combustion flowed rightwards. The hot air moved up along the right wall, then reached the top and moved leftwards. Finally, it was discharged out of the tall space through the upper left opening.

The flow direction of hot air is anticlockwise circulation flow. The upper left opening failed to remove all of the hot air, so that some hot air flowed down on the left side. This phenomenon was recorded obviously in the dynamic photography of Schlieren Photography system, and can also be observed in the simulation results of FDS.

Figure 11 shows that the flow of hot air results in more apparent rotation in the space at 120 s. The temperature in the upper part increased gradually due to the accumulated hot air in the space. In general, in the static picture, the simulated maximum temperature scale of 45 °C can clearly display the flow regime similar to that of the Schlieren image. The scale of 40 °C is more appropriate for dynamic video.

#### Schlieren images and simulation results of Case OT_IL

**Fig. 12:**
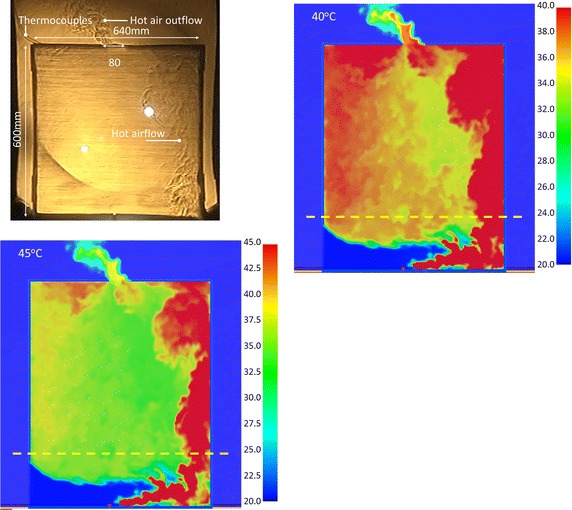
The verification of the Schlieren image and simulation results for CASE OT_IL at 90 s

**Fig. 13:**
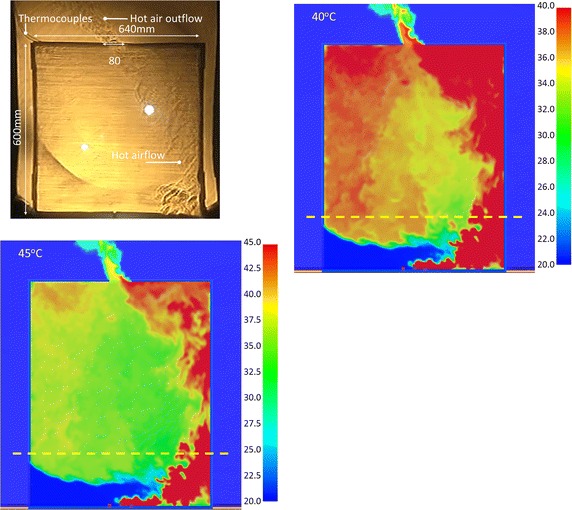
The verification of the Schlieren image and simulation results for CASE OT_IL at 120 s

Figures 12 and 13 compare the hot air of the Schlieren image and numerical simulation in Case OT_IL. The maximum scales were set as 40 and 45 °C for hot air imaging; the simulation results were similar to the Schlieren image. At 90 s, the image of Fig. 12 shows that the external air flow enters from the lower left. The hot air generated by the combustion moves rightwards. As the opening is in the upper center, the hot air moves up along the right wall and flows leftwards. The flow regime of hot air was still counterclockwise cycle motion, but less obvious than Case OL_IL. In the upper space, the hot air not discharged out of the opening flowed continuously to the left and moved down along the left wall. In the dynamic photography of the Schlieren optical system or the simulation result of FDS, the overall flow phenomenon is clearly recorded. The air flow regime in the tall space can be mastered effectively.

At 120 s, Fig. 13 shows that more hot air cannot be discharged through the opening; the accumulation on the left of the space is more apparent. Therefore, the hot air on the right cannot move smoothly, gradually accumulating in the space. The hot airflow accumulated on the right is more apparent than Case OL_IL, accounting for almost 1/2 of the right space. The results prove that using 40 or 45 °C as the maximum temperature scale will match the Schlieren images.

## Conclusions

Taiwan is hot in summer, and the energy saving effect has gradually attracted more attention; the tall space can combine natural ventilation equipment with smoke exhaust equipment by the stack effect. The tall space possesses large capacity, complicated flow pattern and numerous fire scene variables; if the natural smoke exhaust system design is not well considered, the smoke will not be removed effectively. How to design a suitable smoke control system is an important issue for the fire safety of tall spaces. At present, the natural smoke exhaust system is inspected only by checking the area according to the fire regulations in Taiwan.

This study used Schlieren Photography to visualize the hot air pattern in the model space on fire, which was compared with the simulation of FDS. It is feasible to use Schlieren Photography to record the heat flow in buildings. Since the generally invisible hot air was visualized, the effect of air inlet and outlet locations on the hot air flow were recorded and judged clearly; the heat accumulation could be recorded. A thermocouple was used for measurement; the trend of temperature variations is close with simulation results.

The simulation results were compared with the Schlieren images; when the maximum temperature was 40 to 45 °C, their flow patterns were closest to each other. If the maximum temperature scale is 40 °C, the high-temperature region of dynamic image is displayed clearly. When the maximum temperature scale is 45 °C, the high-temperature region of the static picture is displayed better. This study proves that Schlieren Photography can analyze the hot air flow in the tall spaces directly, instantly and accurately.
